# User perception of endocervical sampling: A randomized comparison of endocervical evaluation with the curette vs cytobrush

**DOI:** 10.1371/journal.pone.0186812

**Published:** 2017-11-06

**Authors:** Manuela Undurraga, Rosa Catarino, Isabelle Navarria, Yasmine Ibrahim, Evelyne Puget, Isabelle Royannez Drevard, Jean-Claude Pache, Jean-Christophe Tille, Patrick Petignat

**Affiliations:** 1 Division of Gynecology, Department of Gynecology and Obstetrics, Geneva University Hospitals, Geneva, Switzerland; 2 Division of Clinical Pathology, Geneva University Hospitals, Geneva, Switzerland; Rudjer Boskovic Institute, CROATIA

## Abstract

**Objectives:**

To evaluate whether the endocervical brush (ECB) is better accepted by patients and health care providers for endocervical evaluation when compared to the endocervical curette (ECC), without a decrease in the quality of sampling.

**Methods:**

Two hundred patients with cervical dysplasia were randomized at the colposcopy clinic of the University Hospital of Geneva into two groups according to technique. Patients and physicians’ preference regarding the technique as well as the quality of samples were assessed. ECB samples were analyzed using both cytological (cell block) and histologic analysis, while ECC samples were analyzed using standard histologic analysis.

**Results:**

Of the 200 patients, 89 were randomized to ECC, 101 to ECB and 10 were excluded due to incomplete information or cervical stenosis. Physicians preferred ECB against ECC, classifying it more frequently as an easy technique (94.1% vs.61.4%, p<0.001). Physicians more frequently evaluated the ECB as little or not uncomfortable for patients (28.7% vs.10.2%, p<0.001), though patients themselves didn’t express a preference for either technique. From a quality standpoint, the brush allowed for a better quality of samples, with a lower rate of inadequate samples (2.0% vs 14.3%, p = 0.002) and greater amount of material.

**Conclusion:**

Endocervical sampling using ECB seems to be easier to perform and provides better quality samples. ECB can therefore be an acceptable alternative to ECC in standard practice.

**Trial registration:**

ClinicalTrials.gov NCT01435590

## Introduction

Cervical cancer is the fourth most common cancer worldwide and the fourth cancer in women by mortality [1]. In 2012, 528,000 newly diagnosed cases and 266,000 deaths from cervical cancer occurred worldwide, the equivalent of 8% of cancer deaths, most of which in developing countries [2]. In developed countries, screening programs have led to a significant decrease in the frequency and mortality of this cancer. In Switzerland approximately 240 women develop cervical cancer and 80 patients die of this disease each year. [3]. Cervical cancer develops from pre-invasive lesions or dysplasia, which arise in squamous and glandular cervical cells. Every year in Switzerland, 5,000 women will be diagnosed with dysplasia ranging from ASC-US to HSIL. The most common location for the development of these dysplasias is the transition zone of the cervix, whose position varies with patients’ age and hormonal status. One of the fundamental steps in the diagnosis of dysplasia and cervical cancer is colposcopy and the evaluation of the endocervical canal. The latter is usually done by endocervical curettage, but due to its high rate of false negative results and important patient discomfort, efforts have been made to find alternative techniques [4–7].

The objective of our study was to compare patient and physician preference for two different sampling techniques of the endocervical canal. Our secondary objective was to compare the adequacy of the specimen obtained by each technique.

## Materials and methods

### Setting and study population

This study was conducted at the Geneva University Hospitals and was approved by the local institutional ethics committee (Comité départemental d’éthique de Maternité-Pédiatrie, Commission central d’éthique de la recherché sur l’être humain) (protocol No 11–029). All French speaking patients, older than 21 years that attended our colposcopy clinic were potentially eligible if endocervical evaluation was indicated following national guidelines [8]. Patients with a history of exposure to DES or hysterectomy and pregnant patients were excluded from the study. Two hundred patients were recruited and signed an informed consent form.

### Study procedure and endocervical sample collection

Two techniques were used for the evaluation of the endocervical canal: the endocervical brush (ECB) (COMBIPLUS^®^ by Trimastek CELL COLLECTOR, Switzerland) and the Novak endocervical curette (ECC). The allocation to the technique used was done by randomization in blocks of 4 with a 1:1 allocation via randomization.com. The sequencewas concealed from the physician that was enrolling and assessing participants. Patients were included in a sequentially numbered order through non see-through sequentially numbered envelopes that were opened by a nurse during the exam, once the physician had posed the indication for endocervical evaluation. Indication for endocervical evaluation was based on local guidelines [8] and was performed in patients in whom the transformation zone was not or only partially visible (T-zone 2 or 3). In addition, colposcopy directed biopsies were performed when clinically indicated by the physician performing the exam and as established by local guidelines [8]. Patient concealement of allocation was continued until the end of the exam, and the type of technique used was revealed only after the patient had completed the necessary questionnaires (see below). The technique used for sampling was established in the protocol. In case of randomization to the EEC, the curette was inserted a maximum 3 times in the endocervical canal, using short, firm strokes from the lower uterine segment down to the external os, circumferentially. The samples were then collected and fixed in Carnoy solution. For patients randomized to ECB, the specimen was collected by taking 12 swipes of the entire length of the endocervical canal while rotating simultaneously the brush clockwise. This specimen was then collected and fixed with Thin Prep. To demonstrate a 20% difference (with 80% power and a risk of type 1 error set at 5%) in the quality of the material obtained with the brush, the sample size calculated was of 180 patients.

### Pathologic analysis and specimen adequacy

Cytological and histological interpretation of the ECB specimens was performed using a “cell block” technique, while histological interpretation alone was performed for the ECC specimens. The adequacy of the specimens was based on the quantity of endocervical cells present (< or ≥ 20 endocervical cells) for cytology, while for histology the quantity (< or ≥ 3 epithelial stripes) and quality (absence or presence of lamina propia) of the material were evaluated.

### Data collection

To evaluate the degree of patient discomfort patients were asked to complete a questionnaire once the exam completed. The main acceptance variables were degree of helplessness, pain (on visual analog scales ranging from 0–10), willingness to undergo the test again, and overall satisfaction. Once the exam was completed, the doctor performing the sampling completed a questionnaire indicating his/her perception of the exam (patient’s pain, technical difficulty performing the exam, doctors acceptability of the exam). The evaluation of the quality of the sample was a subjective assessment based on the quantity of material recollected as perceived by the physician.

### Statistical analysis

Quantitative data are expressed as means and standard deviations, and qualitative data are expressed as percentages, unless otherwise stated. Pearson’s chi-square test, student’s t-test or Mann-Whitney test were used, when appropriate, to identify variables related to patients or doctor’s perception of the procedures, as well as, endocervical histological findings and sampling quality that could differ between the two study groups (ECB vs. ECC). All tests were considered as statistically significant when the p-value was inferior to 0.05. Data was analyzed with a statistical analysis software package (StataCorp.2013. Stata Statistical Software: Release 13. College Station, TX, USA).

## Results

### Participants' characteristics

**Fig 1:**
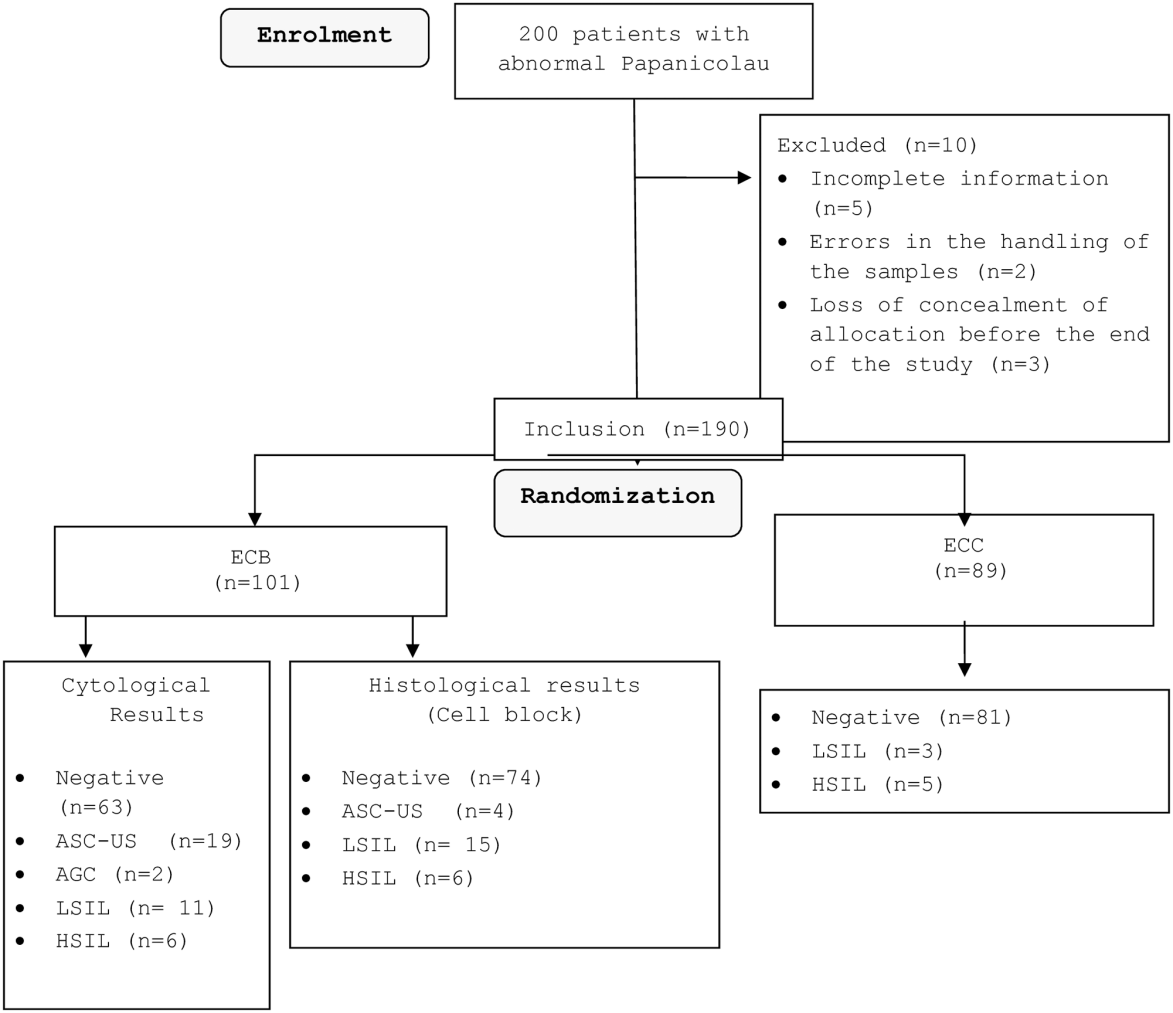
Flowchart of study participants. ECB: endocervical brushing, ECC: endocervical curette.

A total of 200 patients with abnormal Papanicolaou tests were randomized by 13 physicians (ranging from resident to head of the department) to endocervical sampling with either ECC or ECB between September 2011 and October 2014. Ten of the randomized patients did not undergo endocervical sampling and were excluded from the study due to incomplete information (n = 5), errors in the handling of the samples (n = 2) or loss of concealment of allocation before the end of the study (n = 3). Out of the 190 participants, 101 were assigned to ECB and 89 patients to ECC, corresponding to a total response rate of 95%. Fig 1 summarizes the flow chart of study participation. Table 1 summarizes the characteristics of the 190 participants by study group. The median age of women was 34 years in both groups. Twenty-four patients (12%) were menopaused. In the non menopaused group, almost half of the participants didn’t use contraceptive methods.

**Table 1:** Patient characteristics by study group.

Variable	Brushing (n = 101) n.(%)	Curettage (n = 89) n.(%)
**Age, y (median, IQR)**	34 (27–42)	34 (27–41)
**Age categories, y**		
21–30	37 (36.7)	35 (39.3)
31–40	35 (34.7)	29 (32.6)
41–50	15 (14.6)	12 (13.5)
>50	14 (13.9)	13 (14.6)
**Menopause**		
No	90 (89.1)	76 (85.4)
Yes	11 (10.9)	13 (14.6)
**HRT**	0	3 (21.4)
**Contraceptive use**		
No	46 (51.1)	37 (49.3)
Yes	44 (48.9)	38 (50.7)
Not specified	1 (2.3)	2 (5.3)
**Type of contraceptive**		
COC	23 (52.3)	21 (55.3)
Progestogen-only	5 (11.4)	7 (18.4)
Copper IUD	6 (13.6)	4 (10.5)
Hormonal IUD	9 (20.5)	4 (10.5)

Abbreviations: COC = Combined oral contraceptive; HRT = Hormonal replacement therapy; IQR = Interquartile range; IUD = intrauterine device; n. = number; y = years

Numbers may not always add up to 101 for all variables due to missing values

Numbers may not always add up to 89 for all variables due to missing values

### Physician’s appreciation and evaluation of the procedure

According to physicians, ECB was more often classified as the easier technique (94.1% vs.61.4%, p<0.001) and as being able to collect more satisfactory samples for histopathological analysis (62.4% vs.46.6%, p = 0.03) (Table 2) when compared to ECC. Moreover, ECB was evaluated as being little or not uncomfortable at all for patients more frequently than ECC (28.7% vs.10.2%, p<0.001). In average, on a scale of 0 to 10, physician’s perception on the patients’ pain was higher using the curette comparing to the brush (3.3 vs. 2.5, p<0.001). Regarding bleeding, there were no statistically significant differences between the two procedures. Overall, satisfaction was higher with ECB than with ECC, with only one doctor considering brushing as a barely acceptable technique compared to 7 doctors attributing this classification to ECC (p<0.001).

**Table 2:** Doctors' perception and evaluation of each procedure (n = 190).

Variable	Brushing (n = 101) n,(%)	Curettage (n = 89) n.(%)	P value
**How difficult would you classify the procedure?**			**<0.001**
Very easy	62 (61.4)	11 (12.5)	
Easy	33 (32.7)	43 (48.9)	
Acceptable	4 (3.9)	23 (26.1)	
Difficult	1 (1.0)	10 (11.4)	
Very difficult	1 (1.0)	1 (1.1)	
**Technical difficulty grade score (1–5) (mean ± sd)**	1.5±0.72	2.4±0.89	**<0.001**
**How would you describe the presence of bleeding?**			0.177
Absent	15 (14.8)	18 (20.2)	
Very low	33 (32.7)	29 (32.6)	
Low	41 (40.6)	38 (42.7)	
Abundant	12 (11.9)	3 (3.4)	
Very abundant	0	1 (1.1)	
**Specimen adequacy**			**0.03**
Absent or little quantity	38 (37.6)	47 (53.4)	
Satisfactory	63 (62.4)	41 (46.6)	
**How comfortable do you think the patient felt during the procedure?**			**<0.001**
Very uncomfortable	3 (3.0)	5 (5.7)	
Quite uncomfortable	16 (15.8)	35 (39.8)	
Slightly uncomfortable	53 (52.5)	39 (44.3)	
Little or not uncomfortable	29 (28.7)	9 (10.2)	
**How painful do you think the procedure was for the patient (0–10)? (mean ± sd)**	2.5±1.3	3.3±1.6	**<0.001**
**How would you classify your overall satisfaction with the procedure?**			**<0.001**
Very Good	51 (50.5)	11 (12.5)	
Good	43 (42.6)	36 (40.9)	
Acceptable	6 (5.9)	34 (38.6)	
Barely acceptable	1 (1.0)	7 (8.0)	
Unacceptable	0	0	

Abbreviations: n. = number; sd = standard deviation; y = years

Numbers may not always add up to 101 for all variables due to missing values

Numbers may not always add up to 89 for all variables due to missing values

### Patients' acceptability

There were no significant differences between the two randomized groups in relation to patients’ concerns before the procedure. The most reported concern was fear of pain during the exam. Patients’ perception of comfort also didn’t differ between the two groups, with an average score of 2.6 in the ECB vs 3.1 in the ECC (p = 0.062).

The median score of pain reported by physicians vs. patients in each study group; for the ECB group, a median score of 2 was reported by physicians compared to a median score of 3 reported by patients (p = 0.012). In the ECC group, the same median score of pain (3) was reported by both physicians and patients (p = 0.161).

In both groups, the great majority of the patients was satisfied with the procedure and would repeat it in the future (Table 3).

**Table 3:** Patient's acceptability of the procedure by study group.

Variable	Brushing (n = 101) n.(%)	Curettage (n = 89) n.(%)	P value
**How do you find the information received before the study?**			0.339
Very Good	70 (69.3)	52 (59.1)	
Good	21 (20.8)	26 (29.5)	
Acceptable	9 (8.9)	10 (11.4)	
Poor	1 (1.0)	0	
Very poor	0	0	
**What was your level of concern before the exam?**			0.997
Absent	18 (17.8)	16 (18.2)	
Low	28 (27.7)	25 (28.4)	
Moderate	39 (38.6)	34 (38.6)	
High	16 (15.8)	13 (14.8)	
**How comfortable did you feel during the procedure?**			0.464
Very uncomfortable	2 (2.0)	4 (4.5)	
Quite uncomfortable	26 (25.7)	28 (31.8)	
Slightly uncomfortable	48 (47.5)	40 (45.5)	
Little or not uncomfortable	25 (24.8)	16 (18.2)	
**In a scale of 0–10, how painful was the procedure? (mean ± sd)**	3.1±2.1	3.6±2.1	0.062
**Would you consider repeating the exam?**			0.329
Yes	82 (81.2)	68 (78.2)	
No	8 (7.9)	4 (4.6)	
I don't know	11 (10.9)	15 (17.2)	
**How would you classify your overall satisfaction with the procedure?**			0.270
Very Good	41 (40.6)	30 (34.5)	
Good	45 (44.5)	39 (44.8)	
Acceptable	13 (12.9)	18 (20.7)	
Barely acceptable	2 (2.0)	0	
Unacceptable	0	0	

Abbreviations: n. = number; sd = standard deviation; y = years

Numbers may not always add up to 101 for all variables due to missing values

Numbers may not always add up to 89 for all variables due to missing values

### Endocervical histological and cytological analysis

Specimen adequacy analyses results are represented in Table 4. Two samples from the ECB group (2.0%) and 10 samples from the ECC group (14.3%) contained insufficient material for analysis (p = 0.002). There were 97 (98.0%) samples in the ECB group and 74 (86.1%) in the ECC group with presence of epithelial stripes (p = 0.002). In addition, 3 or more epithelial stripes were more frequently present in the ECB group than in the ECC group (87.6% vs. 85.1%, p = 0.047). In the ECB group, among the cases that had an invalid cell block (n = 5), cytology allowed a final diagnosis in 1 case (HSIL, 20%). Inversely, among the 6 cases that had invalid cytology, the cell block, allowed a diagnosis in 50% of cases, all considered normal (Table 5).

**Table 4:** Specimen adequacy by study group.

Variable	Brushing n.(%)	Curettage n.(%)	P value
**Specimen adequacy—Presence of endocervical cells**			**0.002**
Tissue insufficient for diagnosis	2 (2.0)	10 (14.3)	
Amount of tissue for diagnosis satisfactory	96 (98.0)	60 (85.7)	
Number of endocervical cells (cytology only)			
<20	12 (13.0)	NA	
≥20	80 (87.0)	NA	
**Specimen adequacy—Presence of strips (cell block and histology)**			**0.002**
No	2 (2.0)	12 (13.9)	
Yes	97 (98.0)	74 (86.1)	
SOEE (≥3 strips)	85 (87.6)	63 (85.1)	**0.047**
**Specimen adequacy—Presence of lamina propria**			0.223
Yes	38 (38.4)	34 (41.0)	
No	58 (58.6)	42 (50.6)	
Not specified	3 (3.0)	7 (8.4)	

Abbreviations: n. = number; SOEE = Strips of neoplastic epithelium N/A not applicable

Numbers may not always add up to 101 for all variables due to missing values

Numbers may not always add up to 89 for all variables due to missing values

**Table 5:** Pathology agreement between cytology and cell block technique within ECB specimens (n = 101).

		**Cell block**					
		**Normal**	**ASC-US**	**LSIL**	**HSIL**	**Invalid**	**Total**
**Cytology**	**Normal**	**57**	**0**	**0**	**0**	**0**	**57**
	**ASC-US**	**10**	**4**	**3**	**1**	**1**	**19**
	**AGC-NOS**	**1**	**0**	**1**	**0**	**0**	**2**
	**LSIL**	**0**	**0**	**11**	**0**	**0**	**11**
	**HSIL**	**0**	**0**	**0**	**5**	**1**	**6**
	**Invalid**	**3**	**0**	**0**	**0**	**3**	**6**
	**Total**	**71**	**4**	**15**	**6**	**5**	**101**

Abbreviations: ASC-US: Atypical Squamous Cells of Undetermined Significance; AGC-NOS: atypical glandular cells, not otherwise specified; LSIL: Low-grade squamous intraepithelial lesion; HSIL: High-grade squamous intraepithelial lesion.

Of the 178 samples that were adequate for analysis, 46 presented with varying degrees of dysplasia (ASC-US to HSIL). In the ECC group, 7 patients presented with an HSIL (18.4%), 15 patients with an LSIL (39.4%), while in the ECB group, when analyzing the only the cell block technique, 6 patients (16.6%) presented with and HSIL and 15 patients (41.6%) presented with an LSIL.

When analyzing ASC-US, we found that there were 19 cases of ASC-US (50.0%) in the ECB group when analyzed as cytology. When the cell block analysis was added, 73.7% of cases had a final histological diagnosis (52.6% normal, 15.8% LSIL and 5.3% HSIL), while 21.1% were confirmed as ASC-US and 1 case (5.3%) was considered invalid. This means that 55.6% of cases were downgraded and 44.4% were upgraded thanks to the cellblock technique.

The overall agreement between the cytology and the cell block technique in the ECB group was of 72.2%, which gives a Kappa of 0.6 (95% CI 0.40–0.80) and a weighted Kappa of 0.7. This is considered as a “good” strength of agreement. When analyzing the agreement between cytology and cell block in pathological specimens, Kappa was 0.7 (95% CI 0.45–0.92), again considered as a good level of agreement (Table 4).

## Discussion

The evaluation of the endocervical canal is an essential part of the diagnosis and follow-up of patients with cervical dysplasia. The latest recommendations issued by the American Society for Colposcopy [9] highlight the importance of assessing the endocervical canal in almost all women with cervical dysplasia followed by colposcopy. Despite these guidelines, many physicians still hesitate to perform this test frequently. This reluctance is probably based on the fact that this test is often described as painful by patients and doctors. Moreover, in some cases the sample will ultimately prove inadequate because of the absence of sufficient material to perform the analysis, thus hindering the therapeutic and follow-up decision process, with increased costs. The repetition of this test leads to pain and most probably an increase in fear and anxiety, with a consequent decrease in quality of life, as shown in patients with repeat screening mammographies [10]. In view of this situation, it seems important to use the technique that provokes less pain while yielding the best pathological results.

Our study showed a significantly increased rate of adequate endocervical samples with the use of ECB when compared ECC. (98% vs 85.7%, p = 0.002). This might be explained by the double analysis performed with the ECB, both histological using the cell block technique, and cytological. We do not think that this difference in quality is caused by the use of Carnoy instead of formalin for the fixation of our samples. Carnoy was chosen over formolin because it is one of the most effective solutions in the hemolysis of red globules, and is widely used in gynecological samplings, without any artifact when compared to formalin [11].

We also demonstrated that doctors prefer the use of the ECB compared to the ECC when sampling the endocervical canal, finding it easier to manage and that it retrieves more and better material that can then be sent for analysis. They also found the brush to be less painful for patients, but patients did not confirm this observation. It is also interesting to note that most patients, when interrogated on their main fear during the exam, noted “pain” as their main preoccupation, and not “cancer” (52.9% vs 31.7%), which further supports the need for using the least painful sampling method. The process of randomization and the prospective nature of our study are some of the main strengths of the study. This prevented bias caused by choosing patients who could in theory benefit from the ECB technique, such as those with possible cervical stenosis or those who seemed to have a higher degree of stress. Another strength of our study is that, to our knowledge, this is the first study to report physician preference, comparing it to both patients preference and sample quality. The use of disposable material in our study in the ECB group is a third strength of our study, as it follows actual trends in medicine. Last but not least, liquid-based cytology allows reflex HPV testing. As the same technique and materials are used when analyzing the ECB, HPV can also be analyzed in this sample. This is a major point in favor of ECB, since a shift is in action towards cervical cancer screening by HPV [12]. One limitation of the study is the impossibility to assess sensitivity and specificity of each technique due to the absence of a gold standard (histology of conization). We chose to only assess the quality of each sample according to our established protocol assuming that an adequate sample is an essential first step for any histological analysis, and that it is useless to have a very sensitive and specific test if the rate of absence of material is high. The next step in the evaluation of the ECB would be to assess the sensitivity and specificity of each technique using the same analysis criteria, ideally distinguishing between low and high grade lesions. A second limitation of our study is the method of evaluation of acceptability of each technique, which is not objective and based on EVA and visual acceptability scales. None the less, we believe that ultimately the assessment of the acceptability will always be subjective, because based on the emotions of the patients, which must be taken into consideration, regardless of whether they can be objectively quantified or not. A third limitation of our study is based on the inability to perform a double-blinded study. The physicians could obviously see which device they were using. This lack of blinding may have influenced the results in different ways. On one hand, one could question the impact of routine on the physicians’ perception. The ECB is a device used daily by gynecologists for cervical smears. Knowing the smear is usually not painful may have contributed to the perception of a reduction in the intensity of pain in patients sampled by the brush. On the other hand, this familiarity may have allowed doctors to apply more pressure during sampling, improving its quality. Finally, we cannot rule out a "visual effect" on the perception of physicians: a curette, being made of metal, may seem more painful than a basic plastic rod, even before beginning the exam. The final limitation of our study is the absence of evaluation of cervical stenosis in our patients. No patient recruited presented with cervical stenosis prohibiting the completion of the exam. This is probably due to the mean age of our population (34 years), and to the fact that only 12% of our patients were menopaused. One can only hypothize that the presence of stenosis could have influenced both patient and physician perception, as well as the quality of samples, but unfortunately our data does not allow for this analysis. Unfortunately, there is limited literature comparing these techniques for endocervical sampling. This, in addition to the heterogeneity in research hypothesis and study design of previous articles, makes it difficult to compare results. Mogensen [13] conducted a prospected randomized study with patients randomly assigned to either endocervical brush or endocervical curettage. They found that the sensitivity of the cytobrush was higher of that of the endocervical curettage (94% v/s 84% respectively) when combined with biopsies of the ectocervix. The specificities of the two techniques was 95% for the ECB and 88% for the ECC, and 12% of ECC specimens could not be analyzed due to lack of material v/s 0% of the ECB specimens. These results are similar to our own, even though ECB specimens were analyzed only as cytology (not as cell block) and ECC specimens were conserved in formalin. Maksem [4] also found an increase in the quality of ECB samples compared to ECC samples. Unlike our study, his was a retrospective study that analyzed 1507 cases of patients that presented two LSIL or higher colposcopy guided biopsies with ECC and 317 cases that included ECB. As in our study, they performed both cytology and cell block in their ECB specimens. Though no statistical analysis was described, they found that ECB was better than ECC at diagnosing both LSIL and HSIL (16.8% v/s 9.2% respectively for LSIL and 72.2% v/s 63.7% respectively for HSIL). Gibson et al [6] found that ECB specimens were more frequently inadequate when compared to combination ECC followed by ECB or ECC alone (60% v/s 50% v/s 46.9% respectively) and have less stromal strips. This explained by the authors by the less aggressive mechanics during the “scraping”, but this same explanation does not apply to the absence of difference between the 3 groups in either the percent of tissue or the percent of endocervical clusters in the tissue of the specimen. It is interesting to note that in our study we conclude the exact opposite: one of the reasons for the better adequacy in our specimens of the ECB groups is the tendency of the physicians to apply an increased pressure during the procedure due to the perception of decreased pain in this group of patients. Goksedef et al [7] is the final study that found a benefit in the use of ECC compared to ECB. They prospectively randomized 208 patients to either ECB or ECC. Unlike our study, both specimens were analyzed as histology after fixation in formalin. They found similar percentage of inadequate samples (9.5% en the ECC group and 12% in the ECB group), but the ECB group had a statistical significant higher percentage of specimens with no stroma (44% v/s 24% p = 0.003). We think this difference in respect of our study is probably because of the way the ECB specimens were collected and analyzed. The last study published comparing ECB with ECC is by Doo et al [14]. They prospectively evaluated patients who had had an ECB and had to return for an ECC evaluation. They found that there was a very low agreement rate for ECB and ECC in their patients, ranging from 7% in low grade lesions to 16% in high grade lesions. Though this is a very interesting study, there are three possible explanations as to why this agreement is so low. On one hand, one could argue that most patients could have cleared their lesions in the time it took for them to have the repeat exam (0.4 months to 4.7 months). On the other hand, low grade lesions included both LSIL and ASC-US, while high grade lesions included HSIL but also ASC-H. It is known that both ASC-US and ASC-H have a low K value per se, which could have influenced the outcome of the study. Last but not least, since 80% of the ECC samples were negative for disease, it is also possible that small sized lesions were excised during the first endocervical sampling. Though in our knowledge there are no other studies comparing both techniques, other teams, such as Lastra et al [15] and Risse et al [16], have published on the benefit of adding cytology or cell block analysis to ECB and / or ECC samples. Lastra performed cytological analysis of the liquid base transport material left after ECC and found an increase in the diagnostic sensitivity of ECC procedures. Risse on the other hand performed cell blocks in samples positive for AGC, and found an increase in accuracy for the diagnosis of pre invasive or invasive lesions. Both of these results are in line with our findings that the double analysis conducted in the ECB specimens allowed to a higher frequency of adequate when compared to ECC specimens. So far, only two papers have evaluated patient acceptability. Like us, Klam et al [5] found that patients had the same amount of discomfort with both techniques. Unlike us, in their study, the level of patient discomfort perceived by the observer was similar for both techniques, which is in opposition to our findings. We have no explanation for this difference. Goksedef et al [7] on the other hand found that the VSA were significantly different in favor of de ECB group (1.99 v/s 2.55 p<0.001) but the also noted that discomfort was well tolerated with both techniques and no procedure was discontinued because of excessive pain. In their study they do not evaluate physician preference. Mogensen [11] declares in his conclusion that patients preferred the brush to the endocervical curette, which is in line with our results. Unfortunately, we found no information in her article on how this information was obtained or what were the exact results.

## Conclusion

To our knowledge, the present study is the first to compare from a histological point of view the sampling of the endocervical canal by ECB and ECC, while analyzing patient and physician acceptability. In consequence with our results, we believe that the ECB sampling technique of the endocervical canal should be the technique of choice for the quality of sampling and ease of use. A study comparing the samples made with a final pathology cone biopsy could provide pathological confirmation of our findings.

## Supporting information

S1 CONSORT Checklist (DOC)

S1 Research Protocol (PDF)
