# Supplementary material for: User perception of endocervical sampling: A randomized comparison of endocervical evaluation with the curette vs cytobrush
Source: PLoS One. 2017 Nov 6;12(11):e0186812. doi: 10.1371/journal.pone.0186812 (PMC5673173; doi:10.1371/journal.pone.0186812)
Supplement: S1 Research Protocol — (PDF) [file pone.0186812.s002.pdf]

RANDOMIZED COMPARISON OF ENDOCERVICAL EVALUATION WITH THE CURETTE vs CYTOBRUSH FOR THE DIAGNOSIS OF DYSPLASIA OF THE UTERINE CERVIX

Protocol study
February 2011
Version 1.6

Main Investigators
Dr Isabelle Navarria, Service de gynécologie, HUG

Co-investigators
Dr Manuela Undurraga, Service de gynécologie, Ospedale Regionale di Lugano, sede Civico.
Pr Patrick Petignat, Service de gynécologie, Unité d'oncogynécologie chirurgicale, HUG
Pr Michel Boulvain, Service d'obstétrique, Unité de développement, HUG
CC, Dr Marie-Françoise Pelte, service de pathologie clinique, HUG
PD, Dr Jean-Claude Pache, Service de pathologie clinique, HUG

ABSTRACT

OBJECTIVE: to compare endocervical brushing with endocervical curettage with respect to diagnostic and patient discomfort.

METHOD: women referred to colposcopy because of abnormal Papanicolau tests will be randomized to endocervical sampling with either a metal curette (endocervical curettage -ECC) or and endocervical brush. All samples will be submitted for histological study.

EXPECTED RESULTS: higher specimen adequacy and lower patient discomfort with the endocervical brush.

RANDOMIZED COMPARISON OF ENDOCERVICAL EVALUATION WITH THE CURETAGE AND BRUSH FOR THE DIAGNOSIS OF DYSPLASIA OF THE UTERINE CERVIX

INTRODUCTION

In the last decade significant advances in the diagnosis of cervical cancer and pre-invasive disease have been made. One of the fundamental steps in diagnosis is colposcopy with the evaluation of the endocervical canal. This is usually done by endocervical curettage (ECC), but due to its high rate of false positive and false negative results and important patient discomfort, efforts have been made to find alternative techniques.

OBJECTIVE

The objective of this study is to compare the adequacy of specimen of the endocervical brush versus the endocervical curette in the evaluation of the endocervical canal during routine colposcopy done in patients with suspicion of dysplasia. The second end point of this study is to evaluate patient and doctor discomfort with these two methods.

ACTUAL KNOWLEDGE IN THE DOMAIN

In the last decade, no more than 10 original articles have been written comparing endocervical curette with endocervical brush. The design of these studies has been very variable in terms of recollection techniques and analysis of specimen.

Recollection technique

Boardman[1], and Tate[2] used a sequential method for recollection of material, either by using first the curette and then recollecting the material with a brush or the opposite, while Mogensen [3] and Klam [4] used either one or the other. They all found that endocervical sampling with a cytobrush had similar sensitivity and specificity to traditional endocervical curettage. The sensitivity and specificity with

the brush was even higher when using a sleeved cytobrush.

Analysis of specimen

Boardman [1] did a different analysis for the brush and the curette (cytology for the first and histology for the second) while Klam [4], Gibson [5], Maksem [6] and Mogensen [3] all did histological analysis of both samples. This is very important, because it has been demonstrated that when analyzed as cytology, cytobrush technique tends to have high false positive rate. Of all these groups, only Maksem [6] recollected their brush specimen into a liquid fixative process; all others fixed the material onto a slide.

Klam [4] et al evaluated the discomfort associated with each technique, and came to the conclusion that there was no difference.

STUDY DESIGN

This is a prospective randomized study, whose target population is all women consulting our colposcopy clinic. The eligibility of each patient will be analyzed based of the following characteristics:

Inclusion criteria

- French-speaking
- 21 years or older
- Attending our colposcopy clinic
- Need for endocervical evaluation
- Full autonomy or capacity to understand the procedures

Exclusion criteria

- Pregnancy
- Absence of uterus
- History of DES exposure in utero

Information will be given to the patients before commencing their colposcopy, and informed consent will be obtained. Once the colposcopist has decided that a further

evaluation of the endocervical canal is needed, women will then be randomized to either the ECC or the ECB groups. ECC will be performed maximum 3 times with a Novak metal curette using short, firm strokes from the lower uterine segment down to the external os, circumferentially. Samples will then be collected in CARNOY. Endocervical brushing will be performed by taking 12 swipes of the entire length of the endocervical canal while rotating simultaneously the brush. The specimen will then be collected and fixed with Thin Prep. Cytological and histological interpretation of the ECB specimens will then be performed, while for the EEC specimens only histological interpretation will be done. The adequacy of the specimens will be based on the quantity of endocervical cells present (< or ≥ 20 endocervical cells), the quantity of histological material (< or ≥ 3 epithelial stripes) and the quality of histological material (absence or presence of lamina propia).

To evaluate the degree of patient discomfort patients will be requested to complete a questionnaire once the exam has been completed. The main acceptance variables will be degree of helplessness, pain (on visual analog scales), willingness to undergo the test again, and overall satisfaction. Once the exam has been completed, the doctor performing the sampling will answer a questionnaire indicating his/her perception of the exam (patient's pain, technical difficulty performing the exam, doctors acceptability of the exam).

Statistical analysis

The statistical analysis will be based on the comparison of the performance of both tests. The main outcome variable will be the proportion of a satisfactory sample in each group. A satisfactory specimen will be defined by the pathologist as indicated in the paragraph "study design". To demonstrate a 20% difference (with 80% power and a risk of type 1 error set at 5%) in the quality of the material obtained with the brush, we will need a total of 180 patients.

ETHICAL ISSUES

The follow up of patients will not differ from guidelines already in use. The risk of inadequate sampling requiring a second exam has been evaluated as equal for both techniques when analyzing the citology with the slide technique analysis.

FINANCIAL CONSIDERATION

The cost of the different techniques used in this study is 93 francs for the analysis of the material obtained with the brush and 101 to 130 francs for the analysis of the material obtained with the curette, depending on the diagnsosis. Both of these exams will be performed only if indicated during her colposcopic exam, and only one of these techniques will be used. The cost of these exams is covered by normal health insurance, and the actual cost for the patients will depend on their "franchise". The study will include no extra charge for the HUG.

EXPECTED CONTRIBUTION

With this study we hope to demonstrate that endocervical sampling with cytobrush is superior to sampling with endocervical curette in term of diagnosis and patient comfort and doctors acceptability.

COMMENTARY

The main investigators have signed up for the bio-statistic course organized in January 2012. Until that moment, the clinical side of the study will be closely supervised by Prof. P. Petignat, while the biostatistics will be supervised by Prof. M. Boulvain. Both professors have a vast experience in clinical trials, and Prof. Boulvain, has also completed the course in GCP (Good Clinical Practice) in addition to a PhD in epidemiology.

REFERENCES

1. Boardman, L.A., et al., *A randomized trial of the sleeved cytobrush and the endocervical curette.* Obstet Gynecol, 2003. **101**(3): p. 426-30.
2. Tate, K.M. and J.L. Strickland, *A randomized controlled trial to evaluate the use of the endocervical brush after endocervical curettage.* Obstet Gynecol, 1997. **90**(5): p. 715-7.
3. Mogensen, S.T., et al., *Cytobrush and endocervical curettage in the diagnosis of dysplasia and malignancy of the uterine cervix.* Acta Obstet Gynecol Scand, 1997. **76**(1): p. 69-73.
4. Klam, S., et al., *Comparison of endocervical curettage and endocervical brushing.* Obstet Gynecol, 2000. **96**(1): p. 90-4.
5. Gibson, C.A., et al., *Endocervical sampling: a comparison of endocervical brush, endocervical curette, and combined brush with curette techniques.* J Low Genit Tract Dis, 2001. **5**(1): p. 1-6.
6. Maksem, J.A., *Endocervical curetting vs. endocervical brushing as case finding methods.* Diagn Cytopathol, 2006. **34**(5): p. 313-6.
